# Histone hypo-acetylation of *Sox9* mediates nicotine-induced weak cartilage repair by suppressing BMSC chondrogenic differentiation

**DOI:** 10.1186/s13287-018-0853-x

**Published:** 2018-04-10

**Authors:** Kai Tie, Min Wu, Yu Deng, Yinxian Wen, Dan Xu, Liaobin Chen, Hui Wang

**Affiliations:** 1 Department of Orthopedic Surgery, Zhongnan Hospital of Wuhan University, Wuhan, 430071 People’s Republic of China; 2 Department of Pharmacology, Basic Medical School of Wuhan University, Wuhan, 430071 People’s Republic of China; 3 Department of Biochemistry and Molecular Biology, College of Life Sciences, Wuhan University, Wuhan, 430071 People’s Republic of China; 4 Hubei Provincial Key Laboratory of Developmentally Originated Diseases, 185 Donghu Road, Wuchang District, Wuhan, 430071 People’s Republic of China

**Keywords:** Nicotine, Bone marrow-derived mesenchymal stem cells, Chondrogenic differentiation, Cartilage defect, Histone acetylation

## Abstract

**Background:**

Nicotine has negative effects on tissue repair, little research concerns its effect on the cartilage repair of tissue engineering stem cells. The present study aimed to investigate the effects of nicotine on the bone marrow-derived mesenchymal stem cells’ (BMSCs) chondrogenic repair function of cartilage defects and explored the molecular mechanism.

**Methods:**

A cartilage defect model of rat was repaired by BMSC transplantation, and treated with nicotine or saline at 2.0 mg/kg/d in 12 weeks. Nicotine’s effect on chondrogenic differentiation was studied by exposing BMSCs to nicotine at 0.1, 1, 10, and 100 μM, and methyllycaconitine (MLA), which is a selective α7-nicotinic acetylcholine receptor (nAChR) inhibitor and si-RNA of nuclear factor of activated T cells 2 (NFATc2), were used to verify the molecular mechanism of nicotine’s effect.

**Results:**

Data showed that nicotine inhibited cartilage repair function by suppressing SRY-type high-mobility group box 9 (Sox9) in regenerated tissues. Further in vitro study demonstrated that nicotine enhanced intracellular Ca^2+^ and activity of calcineurin (CaN) through α7-nAChR, increased the nucleic expressions of NFATc2 and the bindings to SOX9 promoter, and thus reduced the acetylation of H3K9 and H3K14 in SOX9 promoter.

**Conclusions:**

Findings from this study demonstrated that nicotine suppressed the chondrogenic differentiation of BMSCs in vivo and in vitro, which offers insight into the risk assessment of cartilage defect repair in a nicotine exposure population and its therapeutic target.

**Electronic supplementary material:**

The online version of this article (10.1186/s13287-018-0853-x) contains supplementary material, which is available to authorized users.

## Background

Articular cartilage is composed of chondrocytes and extracellular matrix, which primarily consists of collagen II and proteoglycans [1]. It covers the metaphysis of long bones and provides shock absorption and lubrication to diarthrodial joints. Cartilage tissue has poor self-healing capacity because it is avascular and thus has inadequate access to progenitor cells from blood and bone marrow (BM), which facilitate regeneration [2]. The articular cartilage defect is accompanied by pain and loss of function, and progressive breakdown leads to degenerative disorders, such as osteoarthritis, which can seriously affect a patient’s life and work. Epidemiological studies had indicated that cartilage defects were found in 60% of patients who undergone arthroscopic surgery and that the main population included young adults [3]. Therefore, the optional repair and regeneration of cartilage defect is an important issue in medical research.

Mesenchymal stem cells (MSCs) can be isolated from various tissues and can differentiate into chondrogenic lineage cells in vitro and in vivo [4, 5]. In 2002, Wakitani et al. [6] applied bone marrow mesenchymal stem cell (BMSC) transplantations to repair human articular cartilage defects in osteoarthritic knee joints and observed that the cartilage defects were partially covered with hyaline cartilage-like tissue, which highlighted the availability of autologous culture-expanded BMSC transplantation for the repair of articular cartilage defects in humans. Since then, numerous studies on using BMSCs to repair cartilage lesions, from preclinical findings to clinical application, have been performed as BMSCs can be easily obtained and used [7]. The transplantation of BMSCs has become one of the main methods for treating cartilage defects [8–10]. However, the majority focused on how to optimize the microenvironment or improve the efficiency of chondrogenic differentiation of BMSCs, and there was no concern about adverse factors in the differentiation process.

As a key component of smoking, nicotine has negative effects on tissue repair and healing [11, 12]. An epidemiological study had demonstrated that nicotine abuse was a high-risk factor that influences the clinical outcome of cartilage defects repair of the knee [13]. Our previous study indicated that prenatal nicotine exposure induced fetal articular dyschondroplasia [14].The epiphyseal cartilage development is a process of proliferation and differentiation of MSCs [15], in which nicotine can interfere with the chondrogenic differentiation of MSCs in vivo. The process of repairing cartilage defects is also a process of chondrogenic differentiation of stem cells [16]; therefore, nicotine may have an adverse effect on the chondrogenic repair of BMSCs in cartilage defects.

Our previous work has demonstrated that nicotine could suppress the chondrogenic differentiation potential of BMSCs and result in poorly differentiated cartilage [17]. However, the mechanism is still unclear. SRY-type high mobility group box 9 (*Sox9*) is a key regulator and controls the expression of the α1 chain of type II collagen (*Col2A1*) gene in the initiation stage of MSC chondrogenic differentiation [18]. During primary chondrogenesis, which is activated by transforming growth factor beta (*TGF-β*), *Sox9* binds to essential sequences in the *Col2A1* gene enhancers and initiates its expression [19].The functional modulation of *Sox9* is a critical step for the initiation of chondrogenesis and is mainly regulated by histone acetylation [20]. The nuclear factor of activated T cells (NFAT) transcription factor NFATp (*NFATc2*) is a repressor of chondrogenesis [21] and can interact with histone deacetylase (HDAC) proteins to induce gene silencing [22]. Calcineurin (CaN), an upstream molecule of *NFATc2*, is induced by increased Ca^2+^ and can translocate the dephosphorylated *NFATc2* into the nucleus [23]. The stimulus of nicotine can increase the intracellular Ca^2+^ of MSCs through nicotinic acetylcholine receptor (nAChR) [24]. Therefore, we hypothesized that nicotine could decrease Sox9’s promoter histone acetylation and inhibit its expression through HDAC recruitment by the activated nAChR/Ca^2+^/calcineurin/NFAT pathway in BMSCs.

In this study, we confirmed that nicotine interfered with BMSC transplantation repair of cartilage defects by inhibiting *Sox9* expression in vivo and that nicotine-induced Sox9 gene suppression via nAChRs is mediated directly by decreasing the histone acetylation of the Sox9 promoter.

## Methods

### Animal studies

Animal experiments in this study were approved by the Committee on the Ethics of Animal Experiments of the Wuhan University School of Medicine (No. 14016). The animal experimental procedures were performed in accordance with the Guidelines for the Care and Use of Laboratory Animals (eighth edition) from the National Research Council of the United States National Academies, and performed in the Center for Animal Experiment of Wuhan University (Wuhan, China), which was accredited by the Association for Assessment and Accreditation of Laboratory Animal Care International (AAALAC International). Female (weighing 180–220 g) and male (weighing 260–300 g) Wistar rats at 12 weeks old were obtained from the Experimental Center of the Medical Scientific Academy of Hubei (no. 2008–0005). Rats were housed in metal cages with wire-mesh floors in an air-conditioned room under standard conditions (room temperature: 18–22 °C; humidity: 40–60%; light cycle: 12 h light-dark cycle; 10–15 air changes per hour) and allowed free access to rat chow and tap water.

Rats were randomly distributed into control (BMSCs) and experimental (BMSCs + nicotine) groups (*n* = 8 for each group). The cartilage defect model in the two groups followed the method described by Chung et al. [25]. In brief, anesthesia was performed with an intraperitoneal injection of 3.5 ml/kg of 10% chloral hydrate. In each case, after cleaning with 10% betadine solution, right knee joints of each rat were sterilely draped and opened using an anteromedial approach. The patellae were laterally dislocated, and full-thickness articular cartilage defects (2 mm in diameter) were created in trochlear grooves by carefully drilling in a vertical direction using a 2-mm drill. Drilling was performed in 3 mm deep through subchondral bone. After removing cartilage and bone debris, boundaries around the drill were trimmed using a surgical knife and washed out. The mixture containing BMSCs (2 × 10^6^ cells/ml) and 1.25% alginate in 0.15 M saline [26] was then transplanted into the full-thickness defect in the right knee. Following implantation, patellar retinaculum and overlying soft tissues were closed in layers. Rats were allowed to move knee joints freely in their cages without restriction. An intraperitoneal injection of 4 × 10^5^ U of penicillin (Harbin Pharmaceutical Group, Shanghai, China) was administered immediately after implantation and once daily for 1 week. Clinical signs were observed daily during the study period. Animals were sacrificed at 12 weeks post-implantation to assess cartilage repair statuses. The experimental group was injected with nicotine (Sigma-Aldrich, St Louis, MO, USA) at 1.0 mg/kg twice per day (2.0 mg/kg/d).

As a preliminary study, defects of the same size (2 mm diameter and 3 mm deep) were created, and the cartilage repair with no treatment or alginate only was observed. At 12 weeks post-transplantation, the defects showed very poor cartilage repair (Additional file 1), whose surface was concave, irregular or sometimes empty in the middle and few regenerated tissues could be seen at peripheral regions, meaning that this size of articular cartilage defect in a rat joint is a critical-size defect that cannot heal by itself. Therefore, a defect only without BMSC transplantation (no treatment or alginate only) was not included in the present study.

### Gross and histological evaluations

Rats were sacrificed by cervical dislocation with an intraperitoneal injection of 3.5 ml/kg of 10% chloral hydrate. The entire right knee was dissected, and a distal part of the femur was then extirpated. The samples from each group were examined and photographed to evaluate the degree of defect repair (0–4), integration to border zone (0–4), and macroscopic appearance (0–4) according to the International Cartilage Repair Society (ICRS) macroscopic assessment scale for cartilage repair [27].

After gross examination, samples were fixed in 4% paraformaldehyde, decalcified in 20% ethylenediaminetetraacetic acid (EDTA) (pH 7.4) for 21 d, and embedded in paraffin. Serial 5-mm thick sections were cut in a horizontal plane across the joint. Two sections within every consecutive ten sections were stained with Safranin-O/Fast green (Sigma-Aldrich) and the one with better morphology was used for quantification. The cartilage repair was scored by two blinded authors (K.T. and Y.D.) using a method described by Wakitani [28]. In brief, each section was assigned a score, which was the sum of cell morphology (0–4), matrix-staining (0–3), surface regularity (0–3), thickness of cartilage (0–3), and integration of donor with host (0–3). The expression of Col2A1 and Sox9 in the regenerated tissue was analyzed by immunohistological staining. After antigen retrieval by boiling the samples in sodium citrate buffer, the sections were blocked in serum for 30 min, followed by incubation with the primary antibody in a humidified chamber at 4 °C overnight. A biotinylated secondary antibody was added for 30 min on day 2, followed by an avidin-biotinylated horseradish peroxidase complex, according to the manufacturer’s directions. Finally, the peroxidase activity was revealed by immersion in DAB substrate. The following primary antibodies were used: rabbit anti-Sox9, and rabbit anti-Col2A1 (both from Santa Cruz Biotechnology, Dallas, TX, USA). To characterize the changes in immunostaining, the mean optical densities (MODs) were obtained from ten areas of regenerated tissue from five separate samples.

### Culture of BMSCs and chondrogenic differentiation of BMSCs

The isolation and culture of rat BMSCs from the tibias and femurs of 4-week-old Wistar rats were performed as described previously [17]. Briefly, the rats were sacrificed by cervical dislocation after anesthesia (intraperitoneal injection of 10% chloral hydrate) and sterilized using 75% ethanol for 10 min before surgery. After dissecting the metaphyseal ends of the bones under sterile conditions, the bone marrow cells were flushed out using Dulbecco’s modified Eagle’s medium (DMEM)/F12 medium supplemented with 10% fetal bovine serum (Gibco, Carlsbad, CA, USA), 50 mg/ml of L-ascorbic acid (Sigma-Aldrich), 1% glutamine (Sigma-Aldrich), 100 mg/ml of streptomycin and penicillin (Sigma-Aldrich), and centrifuged at 1000 rpm for 5 min. The cells were resuspended and expanded in T-25 flasks (Cyagen Biosciences, Santa Clara, CA, USA) with DMEM/F12 medium and incubated at 37 °C under conditions of 5% CO_2_. The medium was changed every 3 days. Upon reaching 70%–80% confluence, adherent cells were trypsinized, harvested, and expanded. Cells that had undergone three passages were used in subsequent experiments.

Chondrogenic differentiation of BMSCs was performed following the method described in our previous work [17]. Monolayer culture cells in T-25 flasks were trypsinized, washed, and centrifuged. The isolated BMSCs were suspended at a concentration of 6 × 10^6^ cells/ml in a 1.25% alginate (Sigma-Aldrich) in 0.15 M NaCl, and then the cell suspension was slowly dropped into a 102 mM CaCl_2_ solution. The beads were cultured in six-well plates under conditions of 5% O_2_ with DMEM/F12 medium containing 1% insulin, transferrin and selenous (ITS) (Sigma-Aldrich), 100 nM dexamethasone (Sigma-Aldrich) and 10 ng/ml transforming growth factor-β1 (TGF-β1) (PeproTech Rocky Hill, NJ, USA). During the period of chondrogenic differentiation, the culture medium with or without nicotine at concentrations of 0.1,1,10, and 100μM was replaced every other day. The BMSCs treated with nicotine for 24 h were used in the experiments for molecular mechanism detection.

### RNA interference

siRNAs for *NFATc2* (L-011778) were purchased from Gene Pharma (Shanghai Gene Pharma Co.). The scramble-sense siRNA targeted the sequence 5’-GGAAGCUACAGUGGAUAAATT-3′, and the scramble antisense siRNA targeted the sequence 5’-UUUAUCCACUGUAGCUUCCTT-3′. In brief, passage 3 BMSCs were plated to obtain 70–80% confluence in six-well plates and transfected with si-*NFATc2* using Lipofectamine 2000 (Invitrogen, Carlsbad, CA, USA), negative control siRNA or Lipofectamine 2000 only. After 6 h of transfection, fresh medium was exchanged. The expression of *NFATc2* was detected using RT-qPCR and Western blotting.

### Calcium imaging and phosphatase activity of calcineurin

P3 BMSC cultures in 35 mm glass-bottomed plates were prepared. The cells were washed using Hank’s solution three times and incubated in dye loading solution containing 1μM Fluo-3 AM (DoJinDo, Shanghai, China) at 37 °C for 30 min and then at room temperature for an additional 30 min. Before nicotine stimulation, PBS buffer was used to wash the cells three times. The fluorescence was measured after the addition of 0.1, 1, 10, and 100 μM nicotine. Measurements were performed in a laser scanning confocal microscope (Carl Zeiss, Oberkochen, Germany) by collecting data points every 5 s over 20 min using LCS Lite confocal software.

The phosphatase activity of calcineurin was measured using a calcineurin assay kit (A068, Nanjing Jiancheng Bioengineering Institute, Nanjing, China). In short, the protein of BMSCs was extracted and quantified, the enzymatic reaction was initiated using calmodulin and p-nitrophenyl phosphate (PNPP) as a substrate, and then the inorganic phosphorus content was measured. The enzyme activity unit of calcineurin was defined as the amount of 1 M inorganic phosphorus produced by decomposing 1 mg calcineurin from the substrate PNPP per hour, and the results were expressed as U/mg protein.

### Reverse transcription and real-time quantitative PCR (qRT-PCR)

Total RNA from regenerated tissues and BMSCs was extracted using the Trizol (Invitrogen) reagent following the manufacturer’s protocol. The RNA was reverse transcribed using a first-strand cDNA synthesis kit. The cDNA was amplified using a one-step polymerase chain reaction (RT-PCR) reaction. The PCR products of all of the subtypes of *nAChR* were separated by electrophoresis on 2% agarose gels. The relative mRNA expression levels of the *Sox9* and *Col2A1* were normalized to the level of glyceraldehyde 3-phosphate dehydrogenase (*GAPDH*). The rat primer sequences and annealing temperatures used are shown in Table 1.

**Table 1:** Primer used for qPCR

Gene	Forward Primer	Reverse Primer	Anneal
GAPDH	GCAAGTTCAACGGCACAG	GCCAGTAGACTCCACGACA	60 °C
Col2A1	GAGTGGAAGAGCGGAGACTACTG	CTCCATGTTGCAGAAGACTTTCA	60 °C
Sox9	CCAGCAAGAACAAGCCACAC	CTTGCCCAGAGTCTTGCTGA	60 °C
α3-nAChR	GGTGGATGACAAGACCAAAGC	AGGGCAGGTAGAAGACAAGCA	60 °C
α4-nAChR	CACGGTCTTCGTGCTCAATGT	CCTTGGTTGCAGATGTCACTC	62 °C
α5-nAChR	GCTGCGCTGCTCTTGATGGT	CGTATGTCCACGAGCCGAAT	60 °C
α7-nAChR	ACAATACTTCGCCAGCACCA	GGCATTTTGCCACCATCAGG	60 °C
β2-nAChR	GGAGTGGGAAGATTACCGCCTCA	AGTCGTCGTGGTTCTCGTTGCG	60 °C
β4-nAChR	GATTCTCCCAAGTCAGAACCTTT	AAGCTGGAGATTTGATGTGGTTA	60 °C

*Abbreviations*: *PCR* polymerase chain reaction, *GAPDH* glyceraldehyde phosphate dehydrogenase, *Col2A1* α1 chain of type II collagen gene, *Sox9* SRY-type high mobility group box9, *nAChR* nicotinic acetylcholine receptor

### Western blotting

To obtain total protein, the cells were harvested and dissolved in RIPA (Beyotime, Nanjing, China) buffer. The protein concentrations were determined by a BCA protein assay kit. Equal amounts of protein lysates (40 mg/lane) were loaded and resolved on 10% sodium-dodecyl sulfate-polyacrylamide gel electrophoresis (SDS-PAGE) (Sigma-Aldrich) and then were transferred onto nitrocellulose filters and probed with rabbit anti-Col2A1, Sox9, β-actin, histone 3, NFATc2 (1:900) (all from Santa Cruz Biotechnology), and p-NFATc2 (1:500) (Abcam, Cambridge, MA, USA) antibodies at 4 °C overnight. After incubation with a horseradish peroxidase-conjugated secondary antibody (Santa Cruz Biotechnology), the blots were developed by enhanced chemiluminescence following the manufacturer’s protocol and were visualized by exposure to a Fusion FX system (VilberLourmat, Marne-la-Vallee, France). The protein amounts in electrophoresis gels were analyzed with the Quantity One 4.6 analysis software (Bio-Rad Laboratories, Hercules, CA, USA). All solutions in this procedure contained a mixture of protease and phosphatase inhibitors.

### Chromatin immunoprecipitation assay

Chromatin immunoprecipitation (ChIP) assays were performed as previously described [29]. In brief, 6 × 10^6^ cells were fixed with 1% formaldehyde and quenched by glycine. The cells were washed three times with PBS and then harvested in ChIP lysis buffer (50 mMTris-HCl, pH 8.0, 1% SDS and 5 mM EDTA, all from Sigma-Aldrich). DNA was sonicated to 400–600 bp before extensive centrifugation. Four volumes of ChIP dilution buffer (20 mM Tris-HCl pH 8.0, 150 mM NaCl, 2 mM EDTA and 1% Triton X-100, all from Sigma-Aldrich) were added to the supernatant. The resulting lysate was then incubated with protein G beads (GE Healthcare, Chicago, IL, USA) and H3K9 (Abcam), H3K14 (Abcam) antibodies at 4 °C overnight. The beads were washed five times and DNA was eluted in ChIP elution buffer (0.1 M NaHCO_3_, 1% SDS and 30 μg/ml proteinase K, all from Sigma-Aldrich). The elution was incubated at 65 °C overnight and DNA was extracted with a DNA purification kit (Tiangen Biotech, Beijing, China). The purified DNA was assayed by quantitative PCR assays were repeated at least three times. Data were expressed as a percentage of input DNA. The primer information is in Table 2.

**Table 2:** Primer used for ChIP

Transcript	Sense primer	Antisense primer	Product	Annea
Sox9(327~ 429)	GCTCGGAACTGTCTGGAAAC	GAAACCAGGGCTACTTGCAC	103 bp	60 °C
Col2A1(413~ 546)	GCACCTAGGCGATCTGGTTA	GAGCTGCTGGCTGACTCTTT	134 bp	60 °C

*Abbreviations*: *ChiP* chromatin immunoprecipitation, *Col2A1* α1 chain of type II collagen gene, *Sox9* SRY-type high mobility group box 9

### Immunoprecipitation

Immunoprecipitation was performed following the method described in a previous study [30]. Cell lysates from BMSCs were prepared in NP40 lysis buffer (50 mM Tris-HCl pH 7.4, 150 mM NaCl, 0.5% NP40, all from Sigma) or high-salt lysis buffer (20 mM HEPES pH 7.4, 10% glycerol, 0.35 M NaCl, 1 mM MgCl_2_, 0.5% Triton X-100, 1 mM DTT, all from Sigma-Aldrich) with proteinase inhibitors. The supernatant was then incubated with protein G beads (GE Healthcare) and the NFATc2 antibody (Santa Cruz Biotechnology) at 4 °C for 4 h. Beads conjugated with the lysates and antibodies were collected by centrifugation and washed three times with lysis buffer. The final amount of wash buffer was aspirated and SDS loading buffer was added to the beads. The prepared proteins were resolved using 10% SDS-PAGE and then transferred to nitrocellulose membranes. Finally, the membranes were incubated with antibodies against HDAC1 (Santa Cruz Biotechnology) for 12 h. Chemiluminescence was detected using the abovementioned ECL system.

### Statistical analysis

SPSS 17 (SPSS Science, Chicago, IL, USA) was used for data analysis. Quantitative data were expressed as the means ± SEM. Data of two groups were evaluated with independent sample Student’s *t* tests, and the comparisons among more than two groups were performed using one-way ANOVA followed by Dunnett’s post hoc Student’s *t* tests. The macroscopic and histological score was evaluated using the Mann–Whitney *U* test. Statistical significance was defined as *P* < 0.05.

## Results

### Nicotine had an adverse effect on chondrogenic repair of BMSCs in cartilage defect

**Fig. 1:**
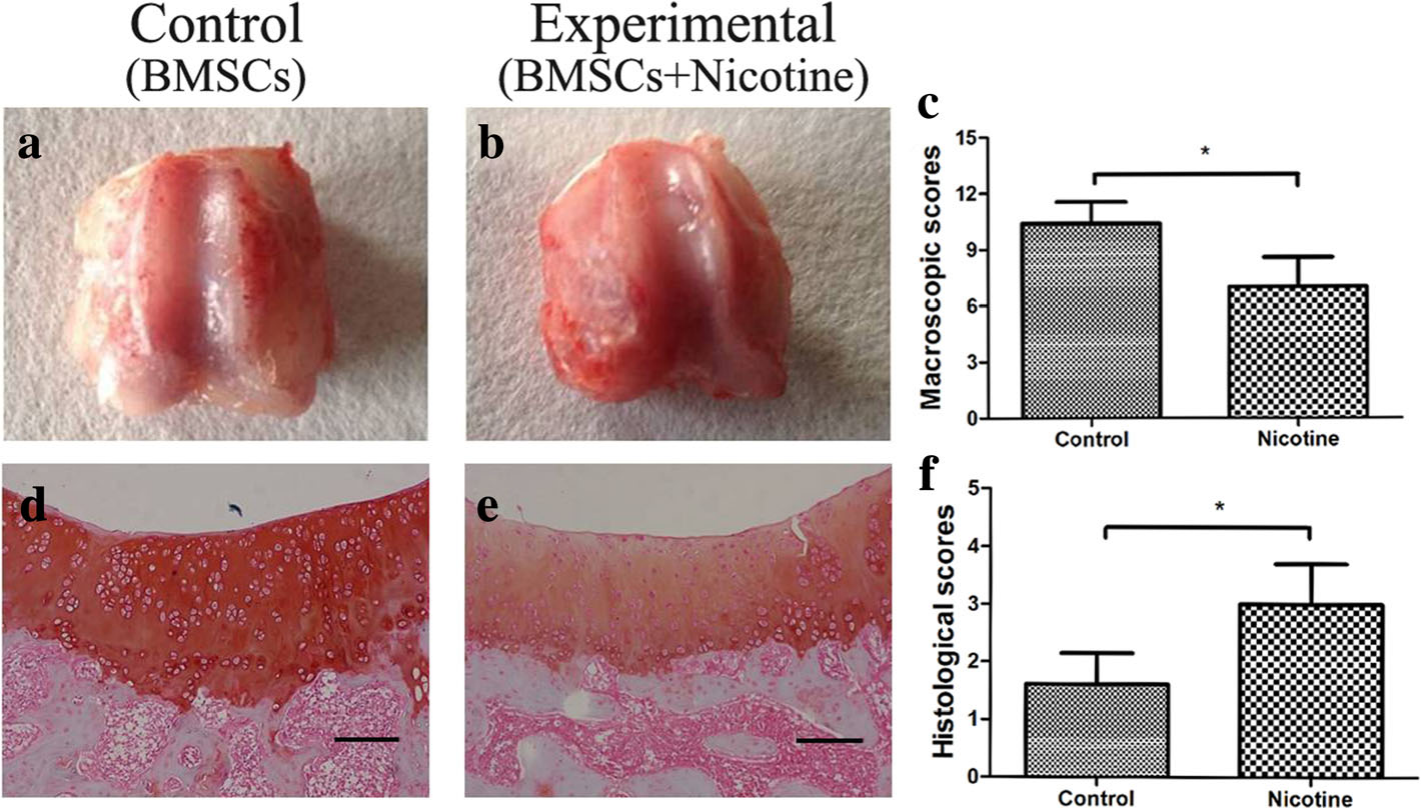
Effects of nicotine on the repair of the cartilage defect with BMSCs transplantation in a rat model after 12 weeks. **a** and **b** Macroscopic observation of cartilage repair (×100). **c** Macroscopic scores of cartilage repair. **d** and **e** Safranin-O and fast green staining of cartilage repair (×100). **f** Histological scores of cartilage repair. Data represent mean ± SD (*n* = 8). *p < 0.05. *Scale bar* = 200 μm. *BMSCs* bone marrow mesenchymal stem cells*.*

To evaluate the impact of nicotine on cartilage defect repair, we used a femoral trochlear cartilage defect rat model, which was repaired by BMSC transplantation. Nicotine was injected at 1.0 mg/kg twice per day (2.0 mg/kg/d). The effect of nicotine on cartilage repair was analyzed. At 12 weeks post-transplantation, no abnormal findings suggested rejection or infection, such as severe inflammation or extensive fibrosis, and no synovial edema was observed in either group. In the control group, the defects were covered with smooth regenerated tissues with surrounding cartilage, and the tissues were well integrated with the surrounding cartilage although there remained part of a depressed tissue with a demarcating border < 1 mm (Fig. 1a). Safranin O staining showed that the cartilage defect was filled to the full depth and smooth regenerated tissues with good column alignment, which were well integrated with both edges of normal cartilage, and the graft had a normal matrix-staining compared with adjacent cartilage. The cell morphology was similar to the native cartilage, which was round or ovoid shaped in neat rows and obvious lacuna (Fig. 1d). In the experimental group, part of the regenerated tissues had a fibrillated surface, most parts of the defects were filled with regenerated tissues level with the surrounding cartilage, little was clearly distinguishable from the normal cartilage, and part of the graft had a fibrillated surface (Fig. 1b). The marked reduced safranin-O staining was exhibited in regenerated tissues, which fully covered the defect. The cell morphology was almost the same as fibrocartilage, which showed an irregular arrangement and no lacuna (Fig. 1e). The macroscopic scores of the control group were much higher than those of the experimental group (Fig. 1c), and the microscopic scores in the control group were much lower than the experimental group (Fig. 1f). The result suggested that the transplantation of BMSCs with alginate could effectively repair the cartilage defect as the regenerated tissue was almost the same as hyaline cartilage. Nicotine had an obvious adverse effect on cell morphology of the regenerated tissue and synthesis of cartilage matrix.

### Nicotine inhibited cartilage repair by suppressing Sox9 expression

**Fig. 2:**
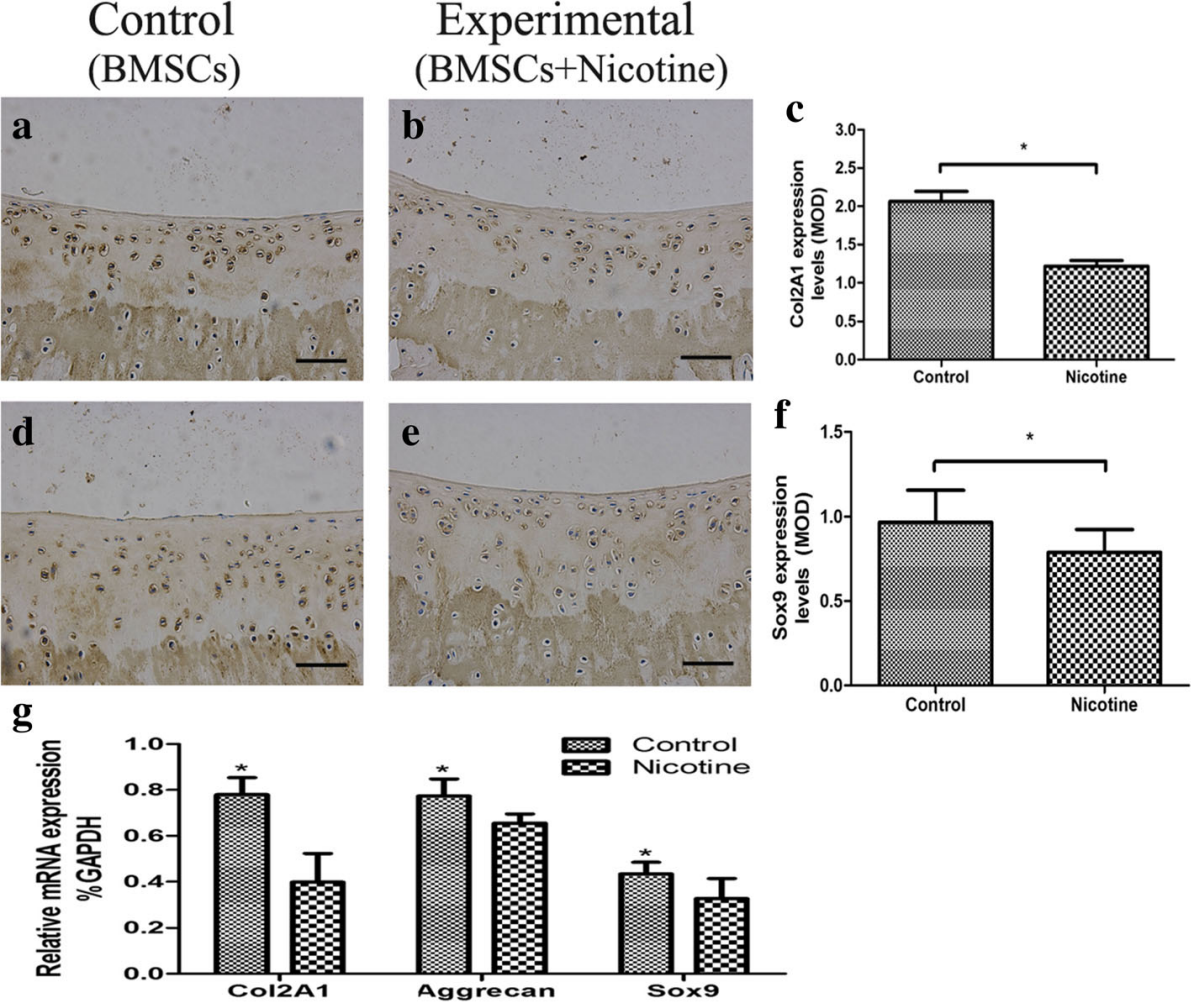
Effects of nicotine on the regenerated tissue of cartilage defect with BMSCs transplantation in a rat model after 12 weeks. **a** and **b** Immunohistological staining of Col2A1 in regenerated tissue (×100). **c** Quantification of the MOD of Col2A1. **d** and **e** Immunohistological staining of Sox9 in regenerated tissue (×100). **f** Quantification of the MOD of Sox9. **g** mRNA expression of Col2A, aggrecan and Sox9 in regenerated tissue. Data represent mean ± SD (n = 8). ^*^p < 0.05. *Scale bar* = 200 μm. *BMSCs* bone marrow mesenchymal stem cells, *Col2A1* α1 chain of type II collagen, *MOD* mean optical density, *Sox9* SRY-type high-mobility group box 9

To explore the mechanism of nicotine’s suppression on the chondrogenic cartilage repair of the cartilage defect, we measured the expression of matrix and Sox9 in the regenerated tissues. Immunohistochemical staining of sections with Col2A1 and Sox9 antibodies showed the proteins in the regenerated tissue stained positive. The staining of Col2A1 was stronger in the control group than the experimental group (Fig. 2a and b), similar to Sox9 (Fig. 2d and e). The mean optical densities of Col2A1 and Sox9 were reduced in the experimental group (Fig. 2c and f). RNA was extracted from the regenerated tissues of both groups, and the mRNA expression of *aggrecan, Col2A1*, and *Sox9* in the experimental group were decreased (Fig. 2g), which was consistent with protein expression by immunostaining. These findings demonstrated that nicotine inhibited the expression of Col2A1 and Sox9 in regenerated tissue. As Sox9 is the upstream cellular signal of *Col2A1*, we concluded that nicotine decreased the expression of *Col2A1* by suppressing Sox9 and then negatively influenced the cartilage defect repair.

### Nicotine increased the intracellular Ca^2+^ and phosphatase activity of calcineurin through α7-nAChR

**Fig. 3:**
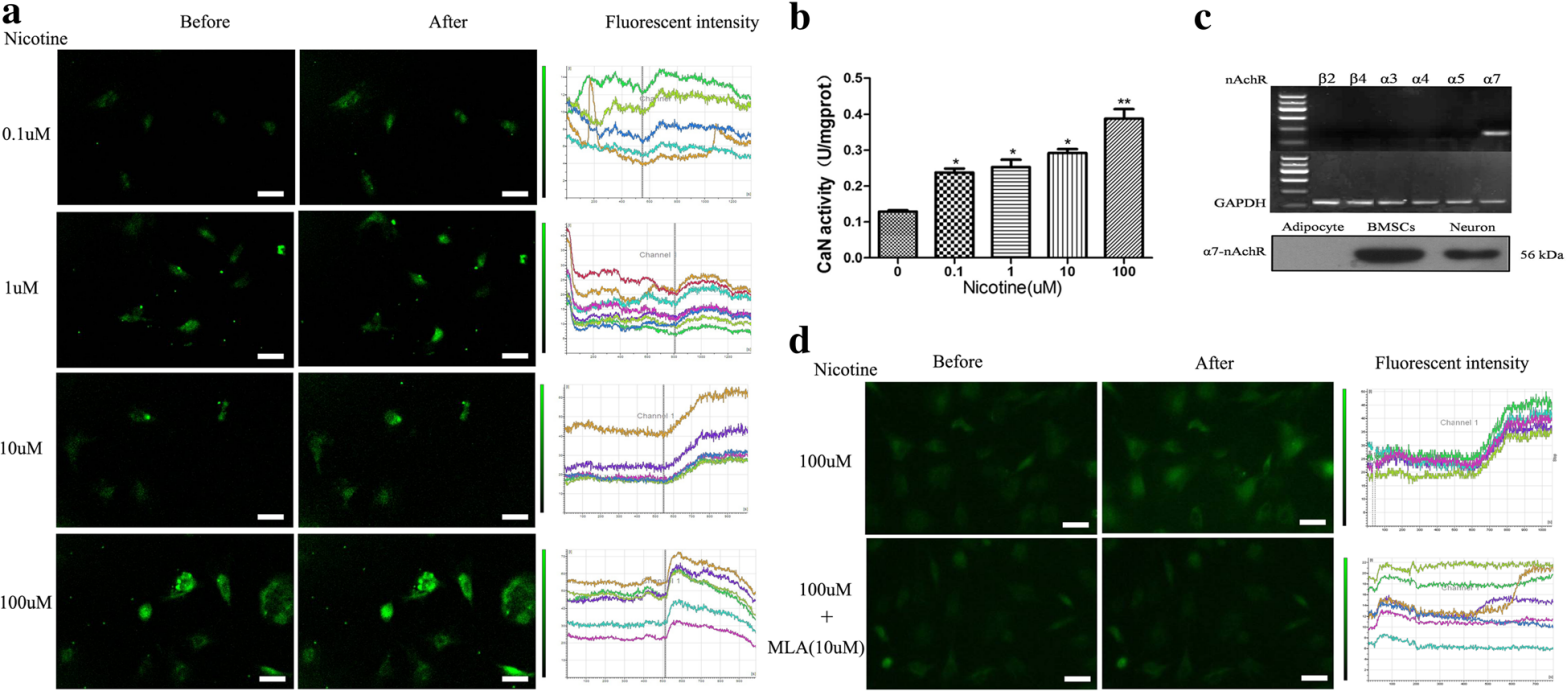
Effects of nicotine on intracellular Ca^2+^ concentration and phosphatase activity of calcineurin in BMSCs. Assay was performed immediately (**a** and **d**) and 30 mins (**b**) after nicotine stimulus of in-vitro culture. **a** Change of fluorescence intensity of Ca^2+^ after the stimulus of nicotine at different concentrations. **b** Change of fluorescence intensity of calcineurin phosphatase activity of CaN calcineurin after the stimulus of nicotine at different concentrations. **c** Expression of the subtypes of nAChR in BMSCs. **d** Effects of MLA on nicotine’s augmentation of intracellular Ca^2+^ concentration in BMSCs. Data represent mean ± SEM (*n* = 3). ^*^p < 0.05, ^**^p < 0.01. *Scale bar* = 100 μm. *BMSCs* bone marrow mesenchymal stem cells, *CaN* calcineurin, *MLA* methyllycaconitine, *nAChR* nicotinic acetylcholine receptor,

To investigate the mechanism of nicotine’s suppression on chondrogenic differentiation of BMSCs, 0.1 to 100μM nicotine treatment was used in BMSCs. The intracellular signals were tested to verify whether the Ca^2+^/CaN/NFAT pathway mediated the inhibitory effect of nicotine. We performed a calcium imaging assay for P3 BMSCs. The results showed that the fluorescence intensity of BMSCs increased 5–10 min after administering nicotine and that the slope of the fluorescence intensity curve was different, which grew gradually with increasing nicotine exposure. Nicotine elicited an increase of intracellular calcium in a concentration-dependent manner (Fig. 3a). The onset time of nicotine in our research was 5–10 min due to the process of nicotine’s diffusion into BMSCs. Thus, we measured the phosphatase activity of calcineurin in 0.5 h after nicotine stimulus and found that the enzyme activity of calcineurin was also increased in a concentration-dependent manner (Fig. 3b).

Because nAChR in MSCs has a large Ca^2+^ permeability and induces elevated intracellular free calcium by releasing intracellular calcium stores, we verified whether nicotine exerted its effect through nAChR in BMSCs. Total RNA and proteins were extracted from BMSCs, and an examination by RT-PCR and Western blotting showed that α7-nAchR was expressed in BMSCs (Fig. 3c). We found that 10μM methyllycaconitine (MLA), the specific antagonist of α7-nAChR, conversely eliminated the nicotine-induced calcium influx (Fig. 3d), implying that the effect of nicotine on BMSCs was fulfilled through α7-nAChR.

### Nicotine suppressed *Sox9* by activating the Ca^2+^/calcineurin/NFATc2 signaling pathway through α7-nAChR

**Fig. 4:**
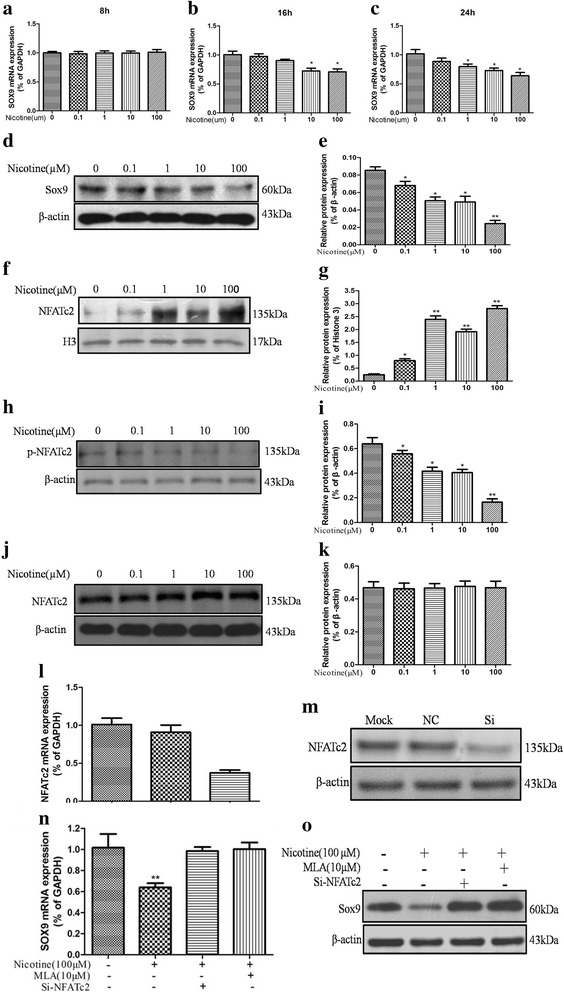
Effects of nicotine on Sox9 and NFATc2 expression in BMSCs. Assay was performed after 24 h of in vitro culture. **a** mRNA expression of Sox9 in BMSCs with stimulus of nicotine for 8 h. **b** mRNA expression of Sox9 in BMSCs with stimulus of nicotine for 16 h. **c** mRNA expression of Sox9 in BMSCs with stimulus of nicotine for 24 h. **d** and **e** Protein expression of Sox9 in BMSCs with stimulus of nicotine for 24 h. **f** and **g** Expression of NFATc2 in nucleoproteins with stimulus of nicotine for 24 h. **h** and **i** Expression of phosphorylated NFATc2 in the cytoplasm with stimulus of nicotine for 24 h. **j** and **k** Expression of NFATc2 in total protein with stimulus of nicotine for 24 h. **l** and **m** Effect of Si-NFATc2 in BMSCs. **n** and **o** Effects of MLA and Si-NFATc2 on nicotine’s suppression of Sox9 in BMSCs. Data represent mean ± SEM (n = 3). ^*^p < 0.05, ^**^p < 0.01. *BMSCs* bone marrow mesenchymal stem cells, *Col2A1* α1 chain of type II collagen, *MLA* methyllycaconitine, *NFATc2* nuclear factor of activated T cell 2, *Sox9* SRY-type high-mobility group box 9

Nicotine exposure increased the concentration of Ca^2+^ and calcineurin phosphatase activity increased over a short period. To detect the molecular mechanism of nicotine’s negative effect, BMSCs were treated with nicotine at different times to detect the expression change of *Sox9*. The results showed that the mRNA expression of *Sox9* decreased in 24 h after nicotine exposure (Fig. 4a-c), and the protein expression of Sox9 also declined (Fig. 4d and e). The NFAT protein has calcineurin binding sites in the regulatory domain and is regulated by Ca^2+^ and calmodulin-dependent serine phosphatase calcineurin [22]; therefore, we extracted the total protein, cytoplasmic proteins and nucleoproteins and found that although no significant changes were observed in the expression of NFATc2 of whole cells (Fig. 4f and g), the expression of nucleic NFATc2 protein was elevated (Fig. 4h and i) and the cytoplasm phosphorylated NFATc2 was reduced by nicotine (Fig. 4j and k), indicating that NFATc2 was rapidly dephosphorylated and localized to the nucleus following nicotine treatment and that the dephosphorylated NFATc2 remains in the nucleus in the continued presence of nicotine. To further verify the molecular mechanism of nicotine’s suppression of *Sox9*, we performed RNAi-mediated stable knockdown of *NFATc2* expression in cultured BMSCs. *NFATc2* expression was significantly suppressed at both the mRNA and protein levels (Fig. 4l and m). The results showed that the expression of *Sox9* had no change when 10 μM MLA and Si-*NFATc2* were given 0.5 h before the nicotine exposure (Fig. 4n and o), implying that interventions aimed at α7-nAChR and *NFATc2* could reverse nicotine’s suppressive effect on the expression of *Sox9*. Therefore, nicotine suppressed *Sox9* by activating the Ca^2+^/calcineurin/*NFATc2* signaling pathway through α7-nAChR.

### Nicotine decreased the histone acetylation on the promoter of *Sox9* in BMSCs

**Fig. 5:**
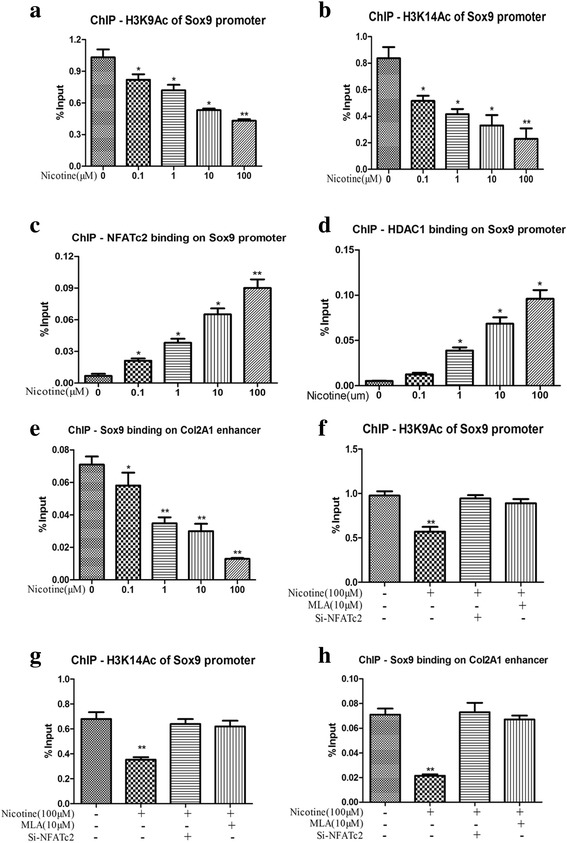
Effects of nicotine on histone acetylation on the promoter of Sox9 in BMSCs. Assay were performed after 24 h of in vitro culture. **a** Change of H3K9 acetylation on the promoter of Sox9. **b** Change of H3K14 acetylation on the promoter of Sox9. **c** Change of NFATc2 binding on the promoter of Sox9. **d** Change of 1 binding on the promoter of Sox9. **e** Change of Sox9 binding on the enhancer of Col2A1. **f** Effects of methyllycaconitine (MLA) and Si-NFATc2 on nicotine’s suppression of H3K9 acetylation on the promoter of Sox9. **g** Effects of MLA and Si-NFATc2 on nicotine’s suppression of H3K14 acetylation on the promoter of Sox9. **h** Effects of MLA and Si-NFATc2 on nicotine’s suppression of Sox9 binding on the enhancer of Col2A1. Data represent mean ± SEM (n = 3). ^*^p < 0.05, ^**^p < 0.01. *BMSCs* bone marrow mesenchymal stem cells, *Col2A1* α1 chain of type II collagen, *HDAC1* histone deacetylase1, *MLA* methyllycaconitine, *NFATc2* nuclear factor of activated T cell 2, *Sox9* SRY-type high-mobility group box 9

Nicotine exposure resulted in the dephosphorylation and increased the nucleic translocation of NFATc2, and then NFATc2 could bind to a site downstream of the transcriptional start site of the target gene and play a role in the downregulation of gene expression [23]. Given the finding that nicotine exposure resulted in a significant decrease of *Sox9* expression, our investigation further focused on the histone acetylation of the *Sox9* promoter. The result of ChIP analysis indicated that the binding of NFATc2 and HDAC1 on the *Sox9* promoter was increased (Fig. 5c and d) and that H3K9ac and H3K14ac levels on the *Sox9* promoter were reduced (Fig. 5a and b) while a decreased binding of Sox9 on *Col2A1* was also observed (Fig. 5e). The above changes induced by nicotine were characterized in a concentration-dependent manner. Moreover, when treatment of 10 μM MLA or si-NFATc2 was performed before nicotine exposure, the levels of H3K9ac and H3K14ac were rescued to the level of untreated control (Fig. 5f and g), and the induced binding of Sox9 on *Col2A1* was also reversed (Fig. 5h). Therefore, nicotine treatment resulted in dephosphorylation and increased nucleic translocation of NFATc2. Then, NFATc2 and HDAC1 bound on the *Sox9* promoter reduced the levels of H3K9ac and H3K14ac on the *Sox9* promoter and decreased *Sox9* expression. Reduced binding of Sox9 with *Col2A1* further decreased *Col2A1* expression.

### Nicotine increased the interaction of NFATc2 and HDAC1

**Fig. 6:**
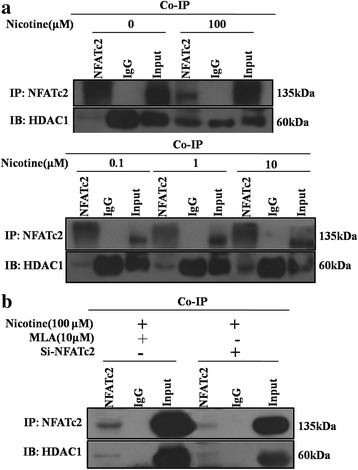
Effects of nicotine on interaction of NFATc2 and HDAC1 in BMSCs. Assay was performed after 24 h of in vitro culture. **a** Interaction of NFATc2 and HDAC1 under stimulus of nicotine with different concentrations. **b** Effects of MLA and Si-*NFATc2* on the interaction of NFATc2 and HDAC1 induced by nicotine. IgG is a negative control. Input is a positive control. NFATc2 is the precipitated protein. HDAC1 is the protein of interest. *BMSCs* bone marrow mesenchymal stem cells, *HDAC1* histone deacetylase1, *MLA* methyllycaconitine, *NFATc2* nuclear factor of activated T cell 2

HDACs participate in decreasing the histone acetylation level of gene promoters. The transcriptional activity of NFAT proteins can regulate gene expression negatively depending on which binding partners are involved. Nicotine exposure resulted in the binding of NFATc2 to Sox9 and decreasing H3K9ac and H3K14ac levels in the Sox9 promoter. To investigate the molecular mechanism of the nicotine-induced decreased level of H3K9ac and H3K14ac in the Sox9 promoter, we used immunoprecipitation to measure the interaction of NFATc2 and HDAC. The result showed that the interaction of NFATc2 with HDAC1 was enhanced in a concentration-dependent manner after nicotine exposure (Fig. 6a), and no significant change of interaction was observed when Si-NFATc2 and MLA were used before nicotine treatment (Fig. 6b). These results implied that NFATc2 could bind to the *Sox9* promoter and recruit HDAC1, which also binds to the *Sox9* promoter to form the complex, and the latter could reduce H3K9ac and H3K14ac levels in the *Sox9* promoter and decrease the expression of *Sox9*.

## Discussion

In the present study, we first verified the adverse effect of nicotine on the chondrogenic repair function of BMSCs in rat articular cartilage defects and proposed that Sox9 plays a key role in this process. Based on our previous study, we further confirmed nicotine’s suppression of *Col2A1* expression and chondrogenic differentiation of BMSCs, the mechanism of which may involve decreasing the histone acetylation of the *Sox9* promoter region through activation of Ca^2+^/CaN/*NFAT* signaling. The altered gene expression and promoter acetylation patterns could be blocked by antagonism of α7-nAChR, indicating that the direct effect of nicotine is mediated by α7-nAChR. Therefore, nicotine could interfere with cartilage repair through suppressing *Sox9* expression in chondrogenic differentiation of BMSCs.

Nicotine is a high-risk factor for many diseases and has negative effects on soft and bony tissue healing [11, 12], which can also influence the repair of cartilage defects and suppress the chondrogenic differentiation of BMSCs [13, 17]. However, the mechanism of nicotine’s adverse effect is still unclear. Previous researches had clarified the chondrogenic potential of MSCs involved in cartilage regeneration [28, 31]. Similarly, the regenerated tissue in our study could have been differentiated from BMSCs. In addition to their differentiation potential, the paracrine action of MSCs was also believed to contribute to their therapeutic effects [32, 33], unknown factors secreted from BMSCs may stimulate chondrogenic differentiation in the repair process. Thus, we believed the mechanism of cartilage repair in defect was associated with chondrogenic differentiation and the secretive action of BMSCs. *Sox9* is a key transcriptional factor and plays an important role in the process of chondrocyte differentiation and cartilage formation [18]. In the initial stage of chondrogenesis, mesenchymal stem cells condense and then differentiate into chondrocytes. *Sox9* can bind to essential sequences in the *Col2A1* gene at chondrocyte-specific enhancers and activate its expression, and *Sox9* has been identified as a regulator of the chondrocyte lineage [34]. Research had shown that the inactivation of *Sox9* in limb buds before mesenchymal condensation resulted in a complete absence of cartilage. A delayed development of cartilage can also be induced when the *Sox9* gene is inactivated after chondrogenic mesenchymal condensation [35]. Thus, *Sox9* is essential in the condensed stage of MSCs and subsequent differentiation in the process of cartilage formation [15]. In addition, *Sox9* also plays an essential role in the physiological control of cartilaginous tissues [36]. BMSCs with *Sox9* gene transfer by adenovirus could promote cartilage defect repair [37]. Therefore, *Sox9* also play an important role in cartilage repair using BMSCs. Our results found that nicotine reduced *Sox9* expression in the regenerated tissue in the cartilage defect area, which demonstrated that nicotine has an adverse effect on cartilage repair through the suppression of *Sox9* in regenerated tissue.

Histone acetyltransferases (HATs) and HDACs are two classes of histone acetylation enzymes, which determine the acetylation status of histones of genes. The activated balance of HATs and HDACs affects the regulation of gene expression [38]. The reduction of acetylation in the transcriptional regulatory regions often leads to the inhibition of gene expression [39]. The Sox9-based transcriptional complex, including *CBP/p300*, which has an intrinsic HATs activity, plays an important role in the initiation of chondrogenic differentiation of MSCs [19]. Thus, the expression of Sox9 itself is mainly regulated by histone acetylation [20]. Histone acetylation is concentrated mainly on histone 3 and histone 4 [40], and H3K9 and H3K14 acetylation often co-occur at many gene regulatory elements and adjust the gene expression in stem cells [41]. Based on these data, we focused on nicotine’s regulation of H3K9 and H3K14 acetylation of Sox9 during the process of chondrogenic differentiation of BMSCs. We observed that nicotine could reduce the expression of Sox9 through decreasing H3K9 and H3K14 acetylation of the Sox9 promoter region and finally suppress the chondrogenic differentiation potential of BMSCs. Our in vitro data are consistent with the in vivo results where nicotine directly decreased the expression of Sox9 through α7-nAChR by suppressing the histone acetylation of the Sox9 promoter during the process of BMSCs chondrogenic differentiation.

NFAT is a family of transcription factors critical in regulating early gene transcription in response to T cell receptor-mediated signals in lymphocytes [42]. *NFATc2* is a repressor of chondrogenesis [21] and can induce gene silencing through interaction with HDAC [22]. Our study demonstrated that *NFATc2* could bind with *Sox9* and recruit HDAC1 to decrease the histone acetylation of the*Sox9* promoter. Calcineurin (also called protein phosphatase 2B), the only serine/threonine protein phosphatase under the control of Ca^2+^/calmodulin, plays a critical role in the coupling of Ca^2+^ signals to cellular responses [43]. Ca^2+^ is the upstream signaling molecule of calcineurin, and the increased Ca^2+^ can activate the enzyme activity of calcineurin. The complex regulation of calcineurin can dephosphorylate phosphorylated serine/threonine proteins and initiate multiple pathways. NFATc2 is the downstream signal of calcineurin [44], and the response to an increase of intracellular Ca^2+^ is dependent upon the dephosphorylation of *NFATc2* by calcineurin. The activated calcineurin controls the translocation of NFAT proteins from the cytoplasm to the nucleus and regulates the expression of target genes [23]. Neuronal nAchR is usually in excitable cells of the central nervous system and controls the influx and efflux of Na^+^, K^+^, and Ca^2+^ [45]. Recent studies found that nAchR was also in non-excitable cells outside the nervous system [24, 46], providing physiologic functions in these cells [47], in which Ca^2+^ plays an important role in a signaling cascade activated by nAchR. Stimulation of MSCs with nicotine induced increases of intracellular Ca^2+^ concentration and initiated the downstream pathway of cascade activation while MLA, which is the specific antagonist of α7-nAChR, could inhibit the calcium influx [48]. In the present study, we found that α7-nAChR was presented in BMSCs and a nicotine stimulus could increase intracellular Ca^2+^ and the enzyme activity of calcineurin of BMSCs. The expression of dephosphorylated NFATc2 in the cytoplasm decreased and *NFATc2* in the nucleus increased. Therefore, nicotine could activate Ca^2+^/Calcineurin/*NFAT* signaling in BMSCs throughα7-nAChR, resulting in recruitment of HDAC1, which decreased histone acetylation of the *Sox9* promoter region and finally inhibited BMSC chondrogenic differentiation.

The dose of nicotine exposure in our study is 2 mg/kg/d, which is consistent with previous animal studies [49, 50]. According to a previous investigation, an average tobacco rod contains 10–14 mg of nicotine, and on average, approximately 1.5 mg of nicotine is absorbed systemically during smoking [51]. Based on the dose–conversion correlation between humans and rats (human: rats = 1: 6.17) [52], the dose of nicotine exposure in the present study is equivalent to the nicotine exposure in an adult weighing approximately 70 kg smoking 2.3 cigarettes per day (calculated as follows: 2 mg/kg/d ÷ 6.17 × 70 kg ÷ 10 mg/cigarette = 2.3 cigarettes/d). The definition of nicotine dependence is the equivalent of more than ten cigarettes per day, and each cigarette contains at least 0.5 mg nicotine [53]. The dose of nicotine in our study was lower than the actual exposure of nicotine dependence in daily life. The present results showed the obvious impact of nicotine on cartilage repair. Thus nicotine’s adverse effect with nicotine dependence would be more serious. The concentration of nicotine (0.01–10 μM) used in our chondrogenic differentiation model is consistent with those often used in pluripotent stem cell studies [54, 55]. The blood concentrations of nicotine during daily smoking are 0.06 μM to 0.23 μM [56], the initial concentration of nicotine in our study was 0.1 μM, which was within the range of a daily smoker’s nicotinic blood concentration and could suppress the chondrogenic differentiation of BMSCs. Based on the principle of toxicology, we set a concentration gradient from 0.1 to 100 μM. The result showed that nicotinic suppression on BMSC chondrogenic differentiation has concentration-dependent characteristics. Although the largest nicotine exposure used in the present study was 100 μM, which was significantly higher than the plasma nicotine level of nicotine dependence, the result was the most obvious and the reversing experiment verified the key point of the molecular mechanism in this concentration, which is also helpful in clarifying the molecular mechanism.

## Conclusions

In this study, we showed that nicotine at a dose under the actual exposure of nicotine dependence in daily life had an adverse effect on the chondrogenic repair of BMSCs in cartilage defects and that nicotine suppressed BMSC chondrogenic differentiation via the activation Ca^2+^/Calcineurin/*NFATc2* signaling, which decreased the histone acetylation of the *Sox9* promoter region. The current study offers insight into the risk assessment of cartilage defect repair in a nicotine exposure population, advising individuals to avoid the negative impact of nicotine on BMSC cartilage repair to achieve high-quality repair of cartilage tissue.

## Additional file

Additional file 1: Macroscopic observation of cartilage defect with no treatment and alginate transplantation in a rat model after 12 weeks. **a** Cartilage defect with no treatment. **b** Cartilage defect with alginate only. (DOCX 413 kb)

## Abbreviations

BMSCs: Bone marrow mesenchymal stem cells
CaN: Calcineurin
ChIP: Chromatin Immunoprecipitation
Col2A1: α1 chain of type II collagen
DMEM: Dulbecco’s modified Eagle’s medium
EDTA: Ethylenediaminetetraacetic acid
GAPDH: Glyceraldehyde 3-phosphate dehydrogenase
HDAC: Histone deacetylase
ITS: Insulin, transferrin and selenous
MLA: Methyllycaconitine
MSCs: Mesenchymal stem cells
nAChR: Nicotinic acetylcholine receptor
NFATc2: Nuclear factor of activated T cell 2
PNPP: P-nitrophenyl phosphate
qRT-PCR: Quantitative real-time polymerase chain reaction
RT-PCR: Polymerase chain reaction
SDS-PAGE: Sodium-dodecyl sulfate-polyacrylamide gel electrophoresis
Sox9: SRY-type highmobility group box 9
TGF-β1: Transforming growth factor-β1
