# Supplementary material for: Histone hypo-acetylation of Sox9 mediates nicotine-induced weak cartilage repair by suppressing BMSC chondrogenic differentiation
Source: Stem Cell Res Ther. 2018 Apr 10;9:98. doi: 10.1186/s13287-018-0853-x (PMC5891899; doi:10.1186/s13287-018-0853-x)
Supplement: Supplementary file 1 — Macroscopic observation of cartilage defect with no treatment and alginate transplantation in a rat model after 12 weeks. a Cartilage defect with no treatment. b Cartilage defect with alginate only. (DOCX 413 kb) [file 13287_2018_853_MOESM1_ESM.docx]

**
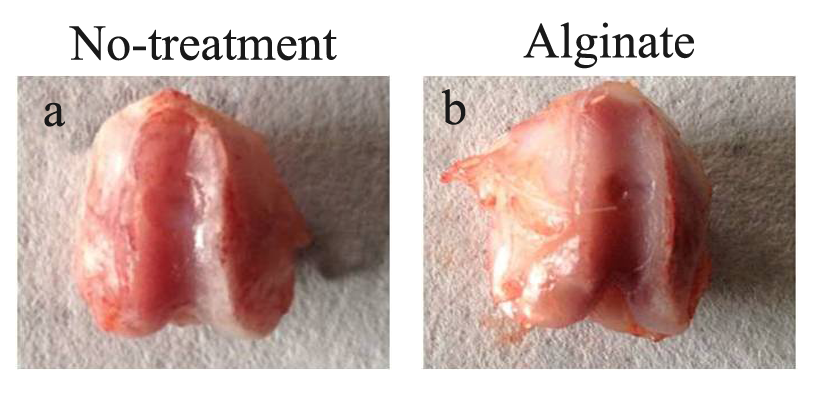
**

**Figure legend**

Macroscopic observation of cartilage defect with no treatment and alginate transplantation in a rat model after 12 weeks (×100). **a** Cartilage defect with no treatment. **b** Cartilage defect with alginate only.
